# Outcomes of patients supported by mechanical ventilation and their families two months after discharge from pediatric intensive care unit

**DOI:** 10.3389/fped.2024.1333634

**Published:** 2024-01-31

**Authors:** Simon MacDonald, Geneviève Du Pont-Thibodeau, Celine Thibault, Camille Jutras, Nadia Roumeliotis, Catherine Farrell, Laurence Ducharme-Crevier

**Affiliations:** Division of Pediatric Critical Care Medicine, Department of Pediatrics, CHU Sainte-Justine, Université de Montréal, Montréal, QC, Canada

**Keywords:** critical care, critical care outcomes, pediatrics, child, follow-up studies, quality of life

## Abstract

**Introduction:**

The outcomes of children undergoing mechanical ventilation (MV) in a Pediatric Intensive Care Unit (PICU) remain poorly characterized and increasing knowledge in this area may lead to strategies that improve care. In this study, we reported the outcomes of children receiving invasive mechanical ventilation (IMV) and/or non-invasive ventilation (NIV), 2 months after PICU discharge.

**Methods:**

This is a post-hoc analysis of a single-center prospective study of PICU children followed at the PICU follow-up clinic at CHU Sainte-Justine. Eligible children were admitted to the PICU with ≥2 days of IMV or ≥4 days of NIV. Two months after PICU discharge, patients and families were evaluated by physicians and filled out questionnaires assessing Quality of life (Pediatric Quality of Life Inventory^™^), development milestones (Ages and Stages Questionnaire), and parental anxiety and depression (Hospital Anxiety and Depression Scale).

**Results:**

One hundred and fifty patients were included from October 2018 to December 2021; 106 patients received IMV (±NIV), and 44 patients received NIV exclusively. Admission diagnoses differed between groups, with 30.2% of patients in the IMV group admitted for a respiratory illness vs. 79.5% in the NIV group. For the entire cohort, QoL scores were 78.1% for the physical domain and 80.1% for the psychological domain, and were similar between groups. Children with a respiratory illness exhibited similar symptoms at follow-up whether they were supported by IMV vs. NIV. For developmental outcomes, only 22.2% of pre-school children had normal scores in all ASQ domains. In the entire cohort, symptoms of anxiety were reported in 29.9% and depression in 24.6 of patients%

**Conclusions:**

PICU survivors undergoing mechanical ventilation, and their families, experienced significant morbidities 2 months after their critical illness, whether they received IMV or NIV. Children with respiratory illness exhibited a higher prevalence of persistent respiratory difficulties post PICU, whether they underwent IMV or NIV. Patients’ quality of life and parental symptoms of anxiety and depression did not differ according to the type of respiratory support. These findings justify the inclusion of patients receiving NIV in the PICU in follow-up assessments as well as those receiving IMV.

## Introduction

One third of children admitted to a Pediatric Intensive Care Unit (PICU) require invasive mechanical ventilation (IMV) (1). Ventilation aims to support respiratory muscles, allow better gas exchange, and reduce oxygen consumption while awaiting recovery from critical illness. Despite its benefits, this life-saving therapy is also associated with several short-term complications, including ventilator-induced lung injury, ventilator-acquired pneumonia, and respiratory muscle atrophy (2). Patients requiring IMV often have a high acuity of illness and are at increased risk of complications such as delirium and withdrawal syndrome, which may impact their recovery trajectory. Non-invasive ventilation (NIV), or mechanical ventilation without intubation or tracheotomy tube, is a method of respiratory support used increasingly as an alternative to IMV, with the goal of minimizing complications (3).

Few studies have assessed the long-term respiratory outcomes of children supported by MV, using either IMV or NIV, during their PICU stay. Two studies evaluated respiratory function in children requiring IMV for acute respiratory distress syndrome (ARDS) three months after their hospitalization. Both found that approximately one-third have persistent respiratory symptoms, and up to 80% have abnormal pulmonary function tests (4, 5). Requiring IMV for a respiratory illness was also associated with a greater risk of subsequently requiring medical care for respiratory issues, with a quarter of patients needing care for another episode of respiratory distress or asthma exacerbation in the 12 months following discharge (6). Long-term data on outcomes of patients requiring IMV for a non-respiratory critical illness are missing. Moreover, data on long-term outcomes other than the respiratory status are lacking. Finally, outcomes of children after NIV are poorly described.

The pediatric post-intensive care syndrome (PICS-p) framework was developed in 2018 to better recognize and assess the new onset or the worsening of impairments arising and persisting after a PICU stay (7, 8). Data on PICS-p are still limited, but a growing literature suggests that significant issues can be appreciated after PICU hospitalization, including developmental delays, post-traumatic stress disorder (PTSD), and a decreased quality of life (QoL) (9–12). The outcomes of mechanically ventilated children who survive a critical illness, regarding the different domains of PICS-p, are still poorly characterized*.* The objective of this study was to report the outcomes of PICU survivors treated with mechanical ventilation 2 months after PICU discharge and to compare outcomes between patients receiving IMV vs. NIV during their PICU stay, with particular emphasis on quality of life.

## Methods

We performed a post-hoc analysis of a single-center prospective study of PICU children followed at the PICU follow-up clinic at CHU Sainte-Justine, a Canadian university-affiliated hospital in Montréal, from October 2018 to December 2021. The local Institutional Review Board approved this study (2019-2261).

### Participants

Patients were identified through the institutional database of the CHU Sainte-Justine (CHUSJ) PICU follow-up clinic. Critically ill patients were eligible for the PICU follow-up clinic if they were less than 18 years old at admission and underwent either IMV for at least 2 calendar days, or NIV for at least 4 days. Patients with congenital heart diseases or active oncologic diseases were not included in the PICU follow-up clinic as they benefit from comprehensive follow-up in other dedicated outpatient clinics. This clinic has been following PICU survivors since fall 2018, and the inclusion criteria were chosen arbitrarily prior to the establishment of the clinic to target a population at risk of PICS-p. In this study, we described this entire cohort of patients with MV, and reported the outcomes of patients who received IMV with or without NIV and patients who exclusively received NIV. Assessment at the 2-month follow-up visit included vital signs and anthropometric measurements, a standardized clinical evaluation by a pediatric critical care physician and the completion of questionnaires by parents and/or patients, as described below.

### Outcomes

Our primary outcome measure was quality of Life (QoL). QoL was assessed with the PedsQL 4.0 Generic Core Scales (≥24 months) and PedsQL Infant Scales (1–24 months) (13, 14). The PedsQL Generic Core Scales is a 23-item measure that evaluates 4 domains (physical, emotional, social, and school) using a 5-point Likert scale (0 = never a problem to 4 = almost always a problem). Total scores range from 0 to 100, with higher scores representing better QoL. Normal values in healthy children [mean ± standard deviation (SD) = 84.1 ± 12.6] have been published (13). The PedsQL Infant Scales assesses 5 domains classified into two categories: 1) physical function and symptoms and 2) emotional, social, and cognitive symptoms. The format and scoring of the Infant Scales are identical to the Generic Core Scales. These two health-related QoL questionnaires have strong reliability and validity in general and specialized pediatrics (15). It is also reliable when filled by proxy for the entire spectrum of pediatric ages. The PedsQL Infant Scales are exclusively completed by parents and the Generic Core Scales are available in both versions (patient and parents).

Secondary outcomes focused on the four domains of PICS-p: physical, cognitive, emotive and social health. Physical health included reported symptoms of dyspnea, voice change, oral aversion, fatigue, weakness, and sleep disorder during physician interview. Cognitive health and developmental delay in preschool children was documented with the Ages and Stages Questionnaires (ASQ-3), a developmental screening tool that assesses developmental stages in children from 1 month to 5 years old through 21 age-specific questions covering five domains (16, 17). Developmental delays were detected by comparing individual scores to determined cut-off scores (18, 19). Obtained scores were then classified into three categories: typical development, mild delay, and moderate-severe delay. For school-aged children, school delay was defined by a change in baseline school performance, assessed during the medical interview. Emotive health was assessed with the Young Child PTSD Checklist (YCPC) in children 1–6 years old (20) and the Child PTSD Checklist (CPC) in children 7–18 years old (21). These tools contain a first section assessing PTSD-related symptoms (avoidance behavior, impaired cognition and mood, and neurovegetative overactivation) and a second section assessing functional impairment. Patients and their parents answer each question on a Likert scale of 0 (never) to 4 (daily). Cutoff values are available for PTSD-related symptoms and functional impairment (21). Finally, parental psychosocial health was assessed through the Hospital Anxiety and Depression Scale (HADS). It is recommended by the National Institute for Health and Care Excellence (NICE) to diagnose anxiety and depression and can also be used to monitor disease progression (22). It includes 7 questions on anxiety and 7 questions on depression (23). Scoring for both categories can point to the absence, probable, or definite presence of symptoms of anxiety and depression. All patients and their family received all the questionnaires with variable response rate.

### Data collection

Data from the PICU hospitalization and follow-up were entered in a case report form, including demographics, pre-PICU comorbidities, and hospitalization-related and follow-up data. Demographic data included age, sex, and weight. Pre-PICU comorbidities potentially associated with QoL or respiratory status included prematurity, airway anomalies, chronic pulmonary disease, and congenital or acquired heart disease. PICU admission data included primary diagnosis, severity of illness as measured by the worst daily Pediatric Logistic Organ Dysfunction Score-2 (PELOD-2) (24), length of IMV, length of NIV, use of vasopressors, and PICU length of stay (LOS). Respiratory disease as the admission diagnosis included children with bronchiolitis, pneumonia, empyema, acute respiratory distress syndrome (ARDS) and bronchospasm. Upper airway diseases included children with laryngitis, tracheitis and subglottic stenosis. All PICU data were manually retrieved from chart reviews. PICU follow-up data were retrieved from the PICU follow-up clinic standardized chart and included breathing difficulties, voice change, oral aversion, cyanosis, fatigue, weakness, sleep disorder and school delay (new onset of academic difficulties).

### Statistical analysis

Analyses were performed with IBM SPSS Statistics (Version 28,0, Armonk, NY). Categorical variables were reported using numbers and percentages. Continuous variables were reported using median (IQR). Comparisons between the IMV ± NIV and NIV exclusively groups were performed using the Pearson chi-square for categorical data and Wilcoxon Mann Whitney for continuous data with non-normal distributions. The level of significance was set to *p* < 0.05.

## Results

We included 150 patients of which 106 patients (71%) received IMV ± NIV (45 IMV only and 61 IMV + NIV) and 44 patients (29%) exclusively received NIV. Demographic and PICU-related data are summarized in Table 1. In this cohort, the mean age was 1.0 year (0.4–3.1), most were male (58.7%) with no previous medical illness (68.0%). The most common admission diagnosis were respiratory illness (44.7%) and septic shock (9.3%). Admission diagnoses differed between groups, with 30.2% of patients in the IMV group admitted for a respiratory illness vs. 79.5% in the NIV group. More patients in the IMV group required the use of vasopressors (39.6% vs. 9.1%).

**Table 1:** Characteristics of PICU patients and ventilation data^a^.

	All patients *N* = 150	IMV ± NIV *N* = 106	NIV *N* = 44
Age (years)	1.0 (0.4–3.1)	1.1 (0.4–6.7)	0.8 (0.4–2.0)
Weight (kg)	8.2 (4.5–15.4)	8.4 (4.3–23.0)	8.1 (5.1–12.4)
Worst PELOD-2	9.0 (4.0–11.8)	10 (8–13)	3 (2–4)
PICU LOS (days)	6.6 (4.5–12.3)	8.0 (4.8–14.0)	5.3 (4.0–8.0)
Follow-up interval (months)	2.3 (1.8–2.7)	2.3 (1.8–3.0)	2.1 (1.7–2.5)
Sex, male (*n*,%)	88 (58.7)	60 (56.6)	28 (63.6)
Pre-admission condition (*n*, %)			
Prematurity	13 (8.7)	7 (6.6)	6 (13.6)
Pulmonary/airway condition	5 (3.3)	1 (1.0)	4 (9.1)
Neurologic condition	16 (10.7)	14 (13.2)	2 (4.6)
Cardiologic condition	4 (2.7)	3 (2.8)	1 (2.3)
Other illness	10 (6.7)	7 (6.6)	3 (6.8)
No known medical illness	102 (68.0)	74 (69.8)	28 (63.6)
Primary admission diagnosis (*n*, %)			
Respiratory illness	67 (44.7)	32 (30.2)	35 (79.5)
Upper airway illness	11 (7.3)	9 (8.5)	2 (4.5)
Traumatic brain injury	13 (8.7)	12 (11.3)	1 (2.3)
Cardiogenic shock	12 (8.0)	9 (8.5)	3 (6.8)
Septic shock	14 (9.3)	13 (12.3)	1 (2.3)
Postoperative	3 (2.0)	3 (2.8)	0 (0)
Neurological illness	7 (4.7)	6 (5.6)	1 (2.3)
Cardiac arrest	4 (2.7)	4 (3.8)	0 (0)
Others	19 (12.7)	18 (17.0)	1 (2.3)
Use of vasopressor (*n*, %)	46 (30.7)	42 (39.6)	4 (9.1)
Ventilation Data, *n* (%)			
Patients requiring invasive mechanical ventilation (IMV)	106 (70.7)	106 (100)	0 (0)
Patients requiring non-invasive ventilation (NIV)	105 (70.0)	61 (57.4)	44 (100)
Length of IMV (days)	3.5 (2.2–7.0)	3.5 (2.2–7.0)	0 (0)
Length of NIV (days)	3.5 (1.0–5.8)	1.6 (0.6–4.6)	4.6 (3.3–6.6)
Total length of ventilation (IMV + NIV)	4.8 (3.0–8.1)	4.9 (2.7–9.6)	4.6 (3.3–6.6)

IMV, invasive mechanical ventilation; NIV, non-invasive ventilation; PICU, pediatric intensive care unit; LOS, length of stay; PELOD-2, pediatric multiple organ dysfunction 2 score.

^a^ Median (IQR) unless otherwise specified.

### Quality of life

Results from the QoL questionnaires are presented in Table 2. A total of 113 families (113/150, 77.4% in the IMV group and 73.8% in the NIV) completed the PedsQL scale, with a majority completed by proxy (92.0%)*.* For the entire cohort, patients reported their QoL at 78.1% for the physical domain and 80.1% for the psychological domain. Overall physical (77.6% vs. 79.7%, *p* = 0.79) and psychosocial (80.0% vs. 80.3%, *p* = 0.72) scores were similar between groups. A similar proportion of children had a score below 1 SD under the mean for the corresponding validated population in both groups for the physical (30/82 patients vs. 9/31 patients, *p* = 0.45) and psychological (19/82 patients vs. 7/31 patients, *p* = 0.95) domains.

**Table 2:** Quality of life score 2 months after discharge from PICU.

	All patients	IMV	NIV	*p*-value
Overall	*N* = 113	*N* = 82	*N* = 31	
Mean physical score, %	78.1	77.6	79.7	0.79
Physical score <1 SD, *n* (%)	39 (34.5)	30 (36.6)	9 (29.0)	0.45
Mean psychosocial score, %	80.1	80.0	80.3	0.72
Psychosocial score <1 SD, *n* (%)	26 (23.0)	19 (23.2)	7 (22.6)	0.95
Completed by patient (8–18 years old)	*n* = 9	*n* = 7	*n* = 2	
Mean physical score, %	85.6	82.4	96.9	0.67
Physical score <1 SD, *n* (%)	1 (11.1)	1 (14.3)	0 (0)	
Mean psychosocial score, %	84.7	82.7	91.7	0.89
Psychosocial score <1 SD, *n* (%)	2 (22.2)	2 (28.6)	0 (0)	
Completed by proxy (2–18 years old)	*n* = 30	*n* = 25	*n* = 5	
Mean physical score, %	80.0	77.1	88.3	0.48
Physical Score <1 SD, *n* (%)	7 (23.3)	7 (26.9)	0 (0)	
Mean Psychosocial score, %	78.5	77.2	85.2	0.45
Psychosocial score <1 SD, *n* (%)	7 (23.3)	7 (26.9)	0 (0)	
Infant (1–12 months)	*n* = 54	*n* = 38	*n* = 16	
Mean physical score, %	78.5	78.6	78.2	0.80
Physical score <1 SD, *n* (%)	22 (40.7)	16 (42.1)	6 (37.5)	
Mean psychosocial score, %	82.9	83.0	82.7	0.90
Psychosocial score <1 SD, *n* (%)	8 (14.8)	5 (13.2)	3 (18.8)	
Infant (13–24 months)	*n* = 19	*n* = 11	*n* = 8	
Mean physical score, %	75.8	77.7	73.1	1.00
Physical score <1 SD, *n* (%)	7 (36.8)	6 (54.5)	1 (12.5)	
Mean psychosocial score, %	72.4	74.3	69.8	0.78
Psychosocial score <1 SD, *n* (%)	9 (47.4)	5 (45.5)	4 (50.0)	

SD, standard deviation; IMV, invasive mechanical ventilation; NIV, non-invasive mechanical ventilation.

### PICS-p domains

**Figure 1:**
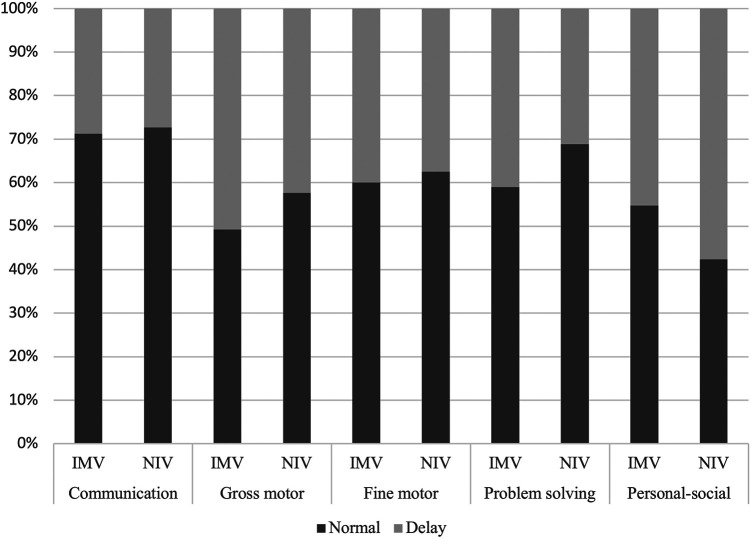
Ages and stages questionnaire score. Results of the ages and stages questionnaire (ASQ) in 16 children, 1–60 months old, evaluating pre-school children's development in five domains. Here presented as typical development (normal) vs. any delay. IMV, invasive mechanical ventilation; NIV, non invasive ventilation.

**Figure 2:**
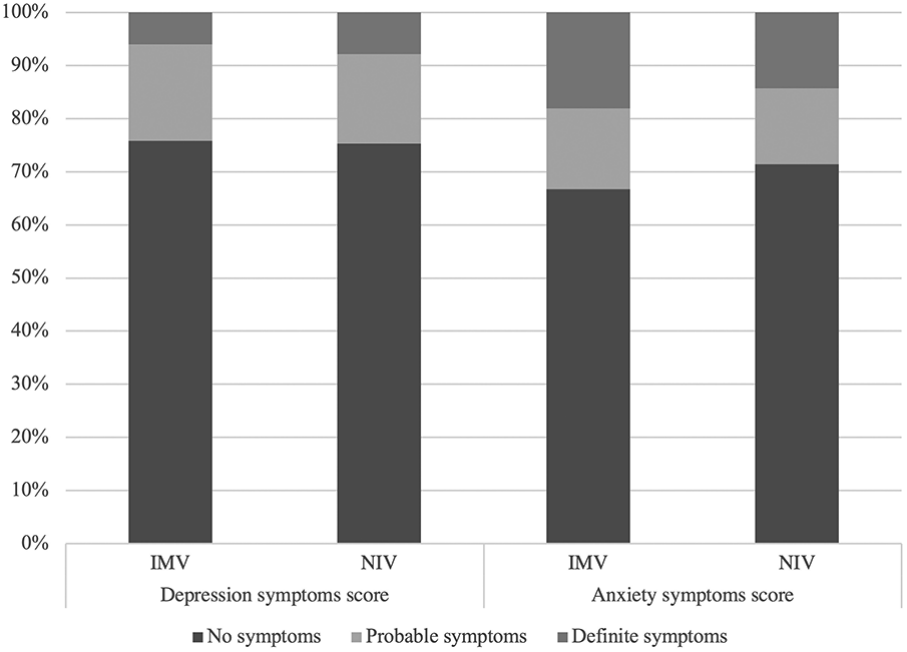
Hospital Anxiety and Depression Scale score. Results of the Hospital Anxiety and Depression Scale, completed by 110 parents. In the IMV group, 16.9% (13/77) reported probable symptoms of depression and 7.8% (6/77) reported definite symptoms of depression whereas 14.3% (11/77) of parents reported probable symptoms of anxiety, 14.3% (11/77) reported definite symptoms of anxiety. In the NIV group, 18.2% (6/33) reported probable symptoms of depression and 6.1% (2/33) reported definite symptoms of depression whereas 15.2% (5/33) of parents reported probable symptoms of anxiety, 18.2% (6/33) reported definite symptoms of anxiety. There was no significant difference in depression scores and anxiety scores between groups (*p* = 0.12 and *p* = 0.31, respectively). IMV, invasive mechanical ventilation; NIV, non-invasive ventilation.

Reported symptoms are shown in Table 3 and data were available for all patients. Regarding the physical domain, fewer patients in the IMV group reported dyspnea (10.4% vs. 27.3%, *p* < 0.01). Fatigue was more common in the IMV group than in the NIV group (18.9% vs. 6.8, *p* = 0.01). There was no difference in voice changes and oral aversion between the two groups. Developmental outcomes were assessed in 99/118 of preschool children (83.9%). Only 22.2% of children had normal scores in all ASQ domains, but there was no difference between groups (Figure 1). Change in baseline school performance was reported in 13/28 (46.4%) and 0/4 (0%) of school-aged children in the IMV and NIV groups, respectively (*p* = 0.07). Emotional domains of PICS-p were assessed in 38 of 75 eligible patients (50.7% of ≥1 year old). In the IMV group, 3/28 (10.7%) of patients reported symptoms of PTSD, and 4/28 (14.3%) reported functional impairments. No patients in NIV group reported symptoms of PTSD or functional impairment (0/10). Finally, parental psychosocial outcomes as assessed through the HADS questionnaire were available for 110 of 150 (73.3%) families. In the entire cohort, symptoms of anxiety were reported in 29.9% and depression in 24.6% These outcomes were similar between the two groups (Figure 2).

**Table 3:** Patients/parents-reported symptoms at 2-months follow-up.

	All patients *N* = 150	IMV ± NIV *N* = 106	NIV *N* = 44	
	*n* (%)	*n* (%)	*n* (%)	*p*-value
Dyspnea	23 (15.3%)	11 (10.4)	12 (27.3)	<0. 01
Cyanosis	2 (1.3)	2 (1.9)	0 (0)	0.36
Voice change	14 (9.3)	11 (10.4)	3 (6.8)	0.50
Fatigue	23 (15.3)	20 (18.9)	3 (6.8)	0.01
Weakness	18 (12.0)	14 (13.2)	4 (9.1)	0.51
Oral aversion	18 (12.0)	10 (9.4)	8 (18.2)	0.13
Sleep disorder	24 (16.0)	19 (17.9)	5 (11.4)	0.23
Learning difficulties	13 (8.7)	13 (12.3)	0 (0)	0.05

IMV, invasive mechanical ventilation; NIV, non-invasive ventilation.

### Admission diagnosis

When comparing IMV patients according to admission diagnosis/indication for IMV, respiratory (32/106) or non-respiratory (74/106), QoL physical (79.0% vs. 76.3%, *p* = 0.87) and psychosocial (82.5% vs. 78.5%, *p* = 0.33) scores were similar. At follow-up, patients who received IMV with a respiratory diagnosis at admission presented more persistent dyspnea (25.0% vs. 4.1%, *p* < 0.01) than those with a non-respiratory diagnosis. However, patients who received IMV for a non-respiratory admission diagnosis reported more fatigue at follow-up (6.3% vs. 24.3%, *p* = 0.03). When comparing patients with a respiratory admission diagnosis who received IMV (24/106) to those who received NIV (26/44), we found no difference in QoL physical (77.2% vs. 78.9%, *p* = 0.90) and psychosocial (81.4% vs. 82.9%, *p* = 0.75) scores. In those children with a respiratory illness, there was no difference in symptoms reported at follow-up in children supported by IMV vs. NIV (Table 4).

**Table 4:** Patients/parents-reported symptoms at 2-months follow-up in patients admitted with a diagnosis of respiratory illness.

	IMV ± NIV *N* = 32	NIV *N* = 35	
	*n* (%)	*n* (%)	*p*-value
Dyspnea	8 (25.0)	11 (31.4)	0.56
Cyanosis	2 (6.3)	0 (0)	0.13
Voice change	3 (9.4)	1 (2.9)	0.26
Fatigue	2 (6.3)	1 (2.9)	0.36
Weakness	2 (6.3)	2 (5.7)	0.99
Oral aversion	4 (12.5)	5 (14.3)	0.83
Sleep disorder	3 (9.4)	3 (8.6)	0.17
Learning difficulties	1 (3.1)	0 (0)	0.57

IMV, invasive mechanical ventilation; NIV, non-invasive ventilation.

## Discussion

This study reports PICS-p related outcomes of a cohort of children undergoing mechanical ventilation in a level-3 PICU, 2 months after discharge. Outcomes of children treated with IMV and those exclusively treated with NIV are also described. Overall, QoL scores of children in our cohort were lower than those of healthy children and developmental delays were common across ventilation groups. PTSD symptoms and functional impairments were present in 10% and 14% of children with IMV, respectively, and were not seen in children with NIV. Parental anxiety and depression were reported in 29.9% and 24.6% of the entire cohort, and present in similar proportions in both groups.

QoL scores were lower than those described in healthy populations (13–15) with 36.0% of the patients studied having scores 1SD below the mean. These results are consistent with a recent study by Watson et al. reporting low QoL scores 6 months post-PICU discharge in children admitted for respiratory failure and receiving IMV (25). A prospective study also reported a persistent decrease in QoL from baseline in 31% of children with ARDS, at 9 months post-discharge (26). Our study is, however, the first to report the outcomes of children receiving MV for a broader variety of indications than respiratory disease.

Persistent dyspnea at follow-up was reported in 15.3%, of all patients, and in 31.4% of children with NIV. When comparing children admitted for a respiratory illness in the 2 groups (IMV ± NIV vs. NIV), there was no difference in respiratory difficulties at follow up. Our findings suggest that children admitted to the PICU for a respiratory illness, irrespective of the mode of ventilation required, are more likely to have residual respiratory symptoms post-PICU and would benefit from medical follow-up. These respiratory symptoms have also been shown to persist over time. For example, a study that investigated the long-term pulmonary outcomes of children less than 2 years old receiving IMV for bronchiolitis reported that a quarter of those children continued to exhibit respiratory symptoms such as asthma once school-aged (27). In terms of other physical symptoms, our study is also the first to report increased fatigue post-PICU in children with IMV. Furthermore, cognitive morbidity was frequent in our cohort, as 83.9% of preschool children exhibited developmental delay and 36.1%. of school age children reported change in baseline school performance after their PICU stay. A recent study, reporting a small cohort of children with normal development at the time of PICU admission for bronchiolitis and supported by high-flow nasal cannula and/or mechanical ventilation, reported that 44% had cognitive disability at 1 or 2 years after PICU discharge (28). Finally, functional decline has been described in 12% of children, 6 months after IMV for bronchiolitis (29). Functionality was not assessed in the current study.

Critical illness and PICU admission both impact on the family of a critically ill child. We detected probable or definite symptoms of anxiety and depression in 29.9% and 24.6% of respondent parents, respectively. The incidence was equally high in parents of children receiving only NIV vs. children with IMV, suggesting that having a child undergoing a non-invasive support may be as distressing to caregivers as having a child who is intubated. This may be due to level of agitation and sedation of the patient, the underlying diagnosis, patient age or a variety of other factors that were not explored in this study.

Our study contributes to the growing body of literature that highlights the extensive range of adverse mid- and long-term outcomes experienced by children admitted to the PICU. It reinforces the impetus for the development of robust and systematic post-PICU follow-up programs. Notably, our research is the first to show that all children, even those requiring NIV without IMV, also experience long-term complications. This underscores the necessity for providing support and follow-up for this group as well. While high illness severity is a recognized risk factor for adverse outcomes, our findings illuminate our incomplete understanding of the complex factors influencing the recovery of both families and their children, encompassing both psychosocial and physical dimensions.

Our study does have some limitations. First, it is a single-center study, and it involved a heterogeneous patient population requiring mechanical ventilation. This heterogeneity may have contributed to a blurring of differences between the two groups, potentially resulting in non-significant findings for some of the outcomes compared. The descriptive nature of this retrospective study also prevents from establishing association between outcomes and mechanical ventilation, as post PICU morbidities described in this study may be secondary to other factors such as pre-existing morbidities and PICU-related exposures (medication, environment, immobilization). This study also has a patient selection bias, as families and patients voluntarily engage into follow-up at our clinic and may not be representative of the entire cohort of patients under mechanical ventilation. Furthermore, it must be noted that only patients undergoing NIV exclusively for at least 4 days were included in this study, and children with shorter duration of NIV were excluded. Consequently, the cohort of patients receiving NIV in this study may experience more severe morbidities than the comprehensive population of patients receiving NIV, as they might suffer from more severe illness and may be exposed for a longer period of time to PICU therapies. Lastly, our follow-up was limited to a relatively short period following the PICU stay and did not include any objective measurement of pulmonary function. Extending follow-up duration could provide a more comprehensive understanding of the issues and the required post-PICU care for this specific cohort. This is especially important considering that certain deficits might improve over time, as noted in previous studies assessing functional impairments (9, 10). Therefore, conducting long-term outcome studies would offer a more nuanced perspective.

## Conclusion

PICU survivors and their families experienced significant morbidities 2 months after their critical illness, whether they received IMV or NIV. Children with respiratory illness exhibited a higher prevalence of persistent respiratory difficulties post PICU, whether they underwent IMV or NIV. Children's QoL and parental anxiety and depression scores were similar irrespective of the type of respiratory support received. These results underscore the importance of extending post-PICU follow-up to include children who received NIV, as they too may benefit from ongoing care and support.

## Data Availability

The raw data supporting the conclusions of this article will be made available by the authors, without undue reservation.
